# Microbial Cross-Talk: Dissecting the Core Microbiota Associated With Flue-Cured Tobacco (*Nicotiana tabacum*) Plants Under Healthy and Diseased State

**DOI:** 10.3389/fmicb.2022.845310

**Published:** 2022-04-14

**Authors:** Waqar Ahmed, Zhenlin Dai, Qi Liu, Shahzad Munir, Jun Yang, Samantha C. Karunarathna, Shichen Li, Jinhao Zhang, Guanghai Ji, Zhengxiong Zhao

**Affiliations:** ^1^College of Resources and Environment, Yunnan Agricultural University, Kunming, China; ^2^State Key Laboratory for Conservation and Utilization of Bio-Resources in Yunnan, Yunnan Agricultural University, Kunming, China; ^3^Key Laboratory of Agro-Biodiversity and Pest Management of Ministry of Education, Yunnan Agricultural University, Kunming, China; ^4^College of Resources, Environment, and Chemistry, Chuxiong Normal University, Chuxiong, China; ^5^Biological Resources Protection and Utilization, College of Biological Resources and Food Engineering, Qujing Normal University, Qujing, China; ^6^College of Agronomy and Biotechnology, Yunnan Agricultural University, Kunming, China

**Keywords:** flue-cured tobacco, disease resistance, locations, microbial diversity, plant components, *Ralstonia solanacearum*

## Abstract

Bacterial wilt caused by *Ralstonia solanacearum* is a devastating disease of flue-cured tobacco production which poses significant yield losses all around the world. In this study, we evaluated the rhizosphere microbiome of healthy and bacterial wilt-infected (diseased) flue-cured tobacco plants through amplification of V3-V4 and ITS1-5f variable regions of 16S and internal transcribed spacer (ITS) rRNA. The study was based on the location (Qujing, Shilin, and Wenshan), plant components (rhizosphere soil and roots), and sample types (healthy and diseased) to assess the diversity of bacterial and fungal communities. Bacterial and fungal communities present in roots primarily emanated from rhizosphere soil. Healthy flue-cured tobacco plants exhibit high microbial diversity compared to diseased plants. Among three variables, plant components significantly influence the diversity of microbial communities, whereas rhizosphere soil harbors higher microbial diversity than roots. Bacterial phyla Cyanobacteria and Proteobacteria were found in high relative abundance in roots and rhizosphere soil samples, respectively. As far as fungi is concerned, a high relative abundance of Ascomycota and Basidiomycota was found in both rhizosphere soil and root. Bacterial genera such as *Bacillus*, *Bradyrhizobium*, *Ensifer*, *Neorhizobium*, and *Lysobacter* related to plant growth promotion and disease suppressing abilities were dominant than fungal genera. Analysis of relative abundance at specie-level revealed that most fungal species are pathogenic to flue-cured tobacco and could provide a conducive environment for wilt infection. In conclusion, *R. solanacearum* significantly influences the microbial diversity of flue-cured tobacco plants and negatively affects the bacterial community composition. Altogether, our study demonstrates the complexity of bacterial and fungal communities that possibly interact with each other (microbe–microbe) and host (host–microbe). This cross-talk could be helpful for healthy flue-cured tobacco plant growth and to induce resistance against bacterial wilt disease.

## Introduction

Bacterial wilt disease caused by soilborne pathogenic bacterium *Ralstonia solanacearum* is a serious threat to flue-cured tobacco (*Nicotiana tabacum* L.) production worldwide, including China (Cai et al., 2021). *Ralstonia solanacearum* is widely distributed in tropical and subtropical regions of the world and has a broad host range that infects more than 250 plant species (Paudel et al., 2020). It is a serious threat to important field crops of the *Solanaceae* family including tomato, potato, tobacco, ginger, eggplant, and pepper, with average yield losses that range from 10 to 55% (Kim et al., 2016).

*Ralstonia solanacearum* infects all tobacco plant parts (roots, stalk, and leaves) and generally produces symptoms of yellowing and wilting of leaves, discoloration of xylem vessels, and black necrotic spots on the stem. It multiplies systemically in the xylem vessels followed by death of whole plant (Cai et al., 2021). Infected soil acts as a primary source of inoculum, and the pathogen oozes out in the rhizosphere soil of diseased plants from the roots upon completion of life cycle (Wu et al., 2020). The bacterium survives in soil, water, and plant residues for a longer period as saprophytism in the absence of a specific host plant (Li et al., 2014). It spreads from diseased to healthy plants through rain splashes, irrigation water, and mechanical operations (Qi et al., 2020).

As far as losses caused by this disease are concerned, in China, incidence and yield losses vary from region to region, host to host, climatic condition, and pathogenic strain (Jiang et al., 2017). However, incidence and yield losses are recorded between 15 to 35% but can reach up to 75% and 50–60%, respectively, when disease is present with root rot pathogen *Phytophtora nicotianae* (Jiang et al., 2017). In high humidity and mono-cropping regions, it occurs in epidemic form, and yield losses reach up to 100%. Soil physicochemical properties, climatic conditions, locations, and rhizosphere microbial diversity play an important role on the occurrence of soilborne diseases (Cai et al., 2021).

The plant rhizosphere is considered as one of the most complex ecosystems on earth and hot spot habitat for diverse microbes (Raaijmakers, 2015). Most disease-resistant and developmental mechanisms in plants are directly related to the diversity of rhizosphere microbes (Mendes et al., 2013; Dong et al., 2019). Plant genotype and soil type are the two main factors that are responsible for assembling a healthy rhizosphere microbiome (Dong et al., 2019). Advancements in science and modern sequencing tools made the study of host–microbe interaction easier (Govindasamy et al., 2014; Mhlongo et al., 2018). However, knowledge gaps are still present between host–microbe interactions and their underlying mechanism, which need to be filled (Bulgarelli et al., 2013).

Nowadays, biological control *via* disease suppressive specific endophytes and rhizobacteria such as *Bacillus*, *Lysobacter*, *Streptomyces*, and *Pseudomonas* is considered as practical approach to suppress the incidence of many soilborne diseases including bacterial wilt, *Fusarium* wilt, and clubroot by the mechanism of direct antagonism, reshaping the rhizospheric microbial diversity, and the production of metabolites (Ma et al., 2018; Wu et al., 2020; Zhang et al., 2020a; Wei et al., 2021). The application of bioorganic fertilizer and biochar along with biocontrol agents significantly suppresses the incidence of tobacco bacterial wilt disease (Liu et al., 2013; Zhang et al., 2017; Li et al., 2022). Because of the broad host range, species complexity, wide geographical distribution, and persistent nature, no effective control exists to date against this devastating disease, and it is difficult to completely control the incidence of tobacco bacterial wilt disease (Li et al., 2014; Shen et al., 2018).

Flue-cured tobacco is a major cash crop in Yunnan Province, China. Yunnan is well-known for its unique environment and climatic conditions. It produces high-quality flue-cured tobacco famous for its pure taste, fragrant aroma, and golden color (Li et al., 2019). Yunnan produces about 50% of China’s total tobacco leaf yield, with an annual production of around 750,000 tons and 320,000 ha of agricultural land under tobacco cultivation (Tang et al., 2020). However, *R. solanacearum* poses significant yield losses every year. Thus, to successfully mitigate the bacterial wilt pathogen, it is necessary to understand the population dynamics and distribution of the microbiome in tobacco plants. Therefore, the present study aims to explore the core microbiota (bacteria and fungi) associated with different locations (Qujing, Shilin, and Wenshan), plant components (rhizosphere soil and roots), and nature of plant (healthy and diseased). We hypothesized that this study helps us to establish a model for studying the naturally occurring flue-cured tobacco microbiome to mitigate the incidence of tobacco bacterial wilt disease.

## Materials and Methods

### Sample Collection

**FIGURE 1:**
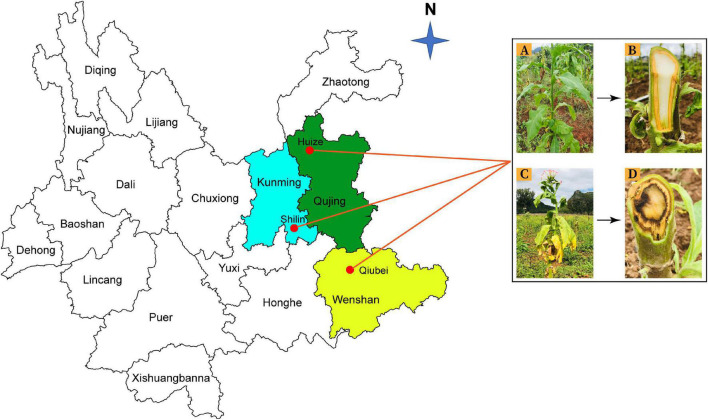
Rhizosphere soil and root samples were collected from three different locations in Yunnan Province: Kunming (Shilin), Qujing (Huize), and Wenshan (Qiubei), from healthy and bacterial wilt infected flue-cured tobacco plants. Healthy flue-cured tobacco plant with green leaves and white-colored xylem vessels **(A,B)**. Disease flue-cured tobacco plant with typical bacterial wilt symptoms (yellowing, wilting of leaves, necrotic lesions on stem, and discoloration of xylem vessels) **(C,D)**.

Rhizosphere soil and root samples were collected from healthy and diseased tobacco plants from three locations; Qujing (25.4900°N, 103.7962°E), Shilin (25.0950°N, 121.5246°E), and Wenshan (23.3863°N, 104.2325°E) in Yunnan Province, China, in September 2020 (Figure 1). Tobacco has been continuously grown in those fields for the past 10 years. For sample collection, upper 2–3 cm layer of soil was removed, and tobacco plants were uprooted (three plants per field from three different fields for both healthy and diseased plants). Bulk soil was removed by shaking the roots, and the tiny soil particles attached to roots were collected as rhizosphere soil samples and fibrous roots as root samples. A total of 12 composite samples (three replicates per sample) were collected from three different locations in Yunnan (Supplementary Figure 1). Samples were put in polythene bags and placed in an icebox until delivered to the laboratory and stored at −80°C for further study.

### DNA Extraction and Polymerase Chain Reaction Amplification

Total genomic DNA was extracted from 0.5 g of soil per sample to 1 g of roots per sample using the Soil and Plant DNA Extraction Kit (Zymo Research Corp., Irvine, CA, United States), respectively, following the manufacturer’s instructions. The quality of extracted DNA was quantified at OD_260/280 nm_ 1.7-1.9 using a NanoDrop spectrophotometer (ND2000, Thermo Scientific, Madison, WI, United States), and extracted DNA was stored at -20°C for future use. The V3-V4 and ITS1-5F variable regions of 16S and internal transcribed spacer (ITS) rRNA genes of bacteria and fungi were amplified using two pairs of universal primers 341F (5′-CCTAYGGGRBGCASCAG-3′) and 806R (5′-GGACTACNNGGGTATCTAAT-3′), and 1743F (5′-GGAAGTAAAAGTCGTAACAAGG-3′) and 2043R (5′-GCTGCGTTCTTCATCGATGC-3′) for bacterial and fungal diversity analysis, respectively (Zhang et al., 2020a).

### Library Preparation and Sequencing

Amplicon library was prepared by the Nextera XT Index Kit (Illumina Inc. Madison, WI, United States) as per 16S and ITS Metagenomic Sequencing Library preparation protocols. Amplicon quality was visualized using gel electrophoresis, and 1X AMPure XP beads were used for amplicons library purification, checked on Agilent DNA1000 chip with Bioanalyzer2100, and quantified by Qubit Fluorometer 2.0 using a Qubit dsDNA assay kit (Life Technologies Cat. No. Q328520) (Gao et al., 2018). The same numbers of purified amplicons were pooled for subsequent sequencing analysis and sequenced on an Illumina MiSeq platform at Novogene Bioinformatics Technology Co. Ltd. (Beijing, China).

### Quality Control

Raw data were collected in FASTQ format from DNA sequencing, and Trimmomatic software was used for the cutoff of low-quality reads (score < 20) and preparation of paired-end reads (Bolger et al., 2014). FLASH software was used to assemble paired-end reads with 10 bp/200 bp and 20% minimum/maximum overlapping and maximum mismatch rate, respectively. UCHIME software was used for chimeras removal and production of clean reads (Edgar et al., 2011).

### Data Processing

Clean reads were processed with the UPARSE pipeline to generate operational taxonomic units (OTUs) at ≥97% similarity level (Edgar, 2013). For taxonomic information, species annotation was performed for all representative read and OTUs using ribosomal database project (RDP) classifier in SILVA database for bacteria (at 70% confidence threshold) and UNITE database for fungi (Quast et al., 2012; Kõljalg et al., 2013). OTUs were analyzed for relative abundance at genus and phylum levels, and alpha and beta diversity indices were calculated to obtain species richness and uniformity information. A Venn diagram was used for common and unique OTUs among different variables such as sample types, plant components, and locations.

### Statistical Analysis

Data were statistically analyzed using a *t*-test (*P* < 0.05). All statistical analyses were performed using IBM SPSS Verison 20.0 (SPSS Inc., Chicago, IL, United States). QIIME software (Version 1.9.1) was used to calculate the observed OTUs, Chao1, Shannon, and abundance-based coverage estimator (ACE) indices. The Bray–Curtis dissimilarity was calculated for beta diversity analysis of bacterial and fungal communities and used for principal coordinate analysis (PCoA) with QIIME. The relative abundance bar plots at the phylum level, relative abundance heatmaps at the genera level, and relative abundance bar plots at the species level were generated using R scripts in R software (version 2.15.3) (Dong et al., 2018). Co-occurrence network analysis was conducted using sparcc in R for OTUs at the phylum level (*P <* 0.05 and correlation coefficient > 0.3). The network properties were calculated and visualized in Gephi 0.9.2. All figures were processed and illustrated using Adobe Illustrator CC 2019 (Adobe Systems Inc., San Francisco, CA, United States).

## Results

### General Characteristics of Tobacco Microbiome

We explored the bacterial and fungal communities associated with different parts of the tobacco plant (rhizosphere soil and roots), different geographic locations (Qujing, Shilin, and Wenshan), and different sample types (healthy and diseased). Data related to raw reads (#), clean reads (#), and quality control (Q20% and Q30%) through amplification of 16S (V3-V4)/ITS (1-5f) rRNA of bacteria/fungi, respectively, are shown in Supplementary Table 1. After quality control and chimeras filtering, an average of 80,477 bacterial and 92,685 fungal clean reads per sample were obtained with an average length of 412 bps/244 bps per sample by Illumina sequencing (Supplementary Table 1). Rarefaction curves generated from the OTUs demonstrated that high sampling coverage was achieved in all samples for both fungal and bacterial communities (Supplementary Figure 2).

### Effects of Different Locations, Plant Components, and Sample Types on Beta Diversity

**FIGURE 2:**
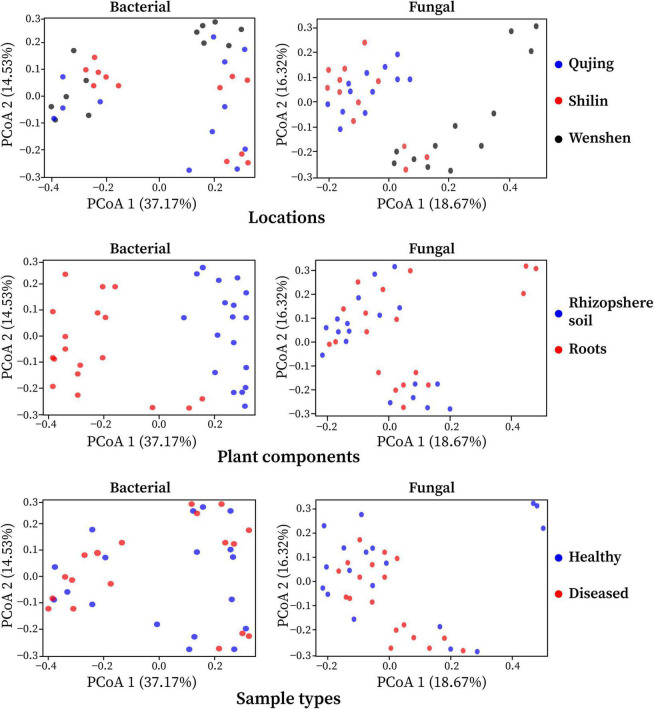
Principal coordinate analysis (PCoA) was based on the Bray–Curtis dissimilarity metrics showing the beta diversity analysis for all 12 composite samples (three replicates per sample) of flue-cured tobacco plants under three variables.

The effect of variables such as locations (Qujing, Shilin, and Wenshan), plant components (rhizosphere soil and roots), and sample types (healthy and diseased) on bacterial and fungal community composition was analyzed. Bray–Curtis dissimilarity was used to determine the beta diversity (variation in bacterial and fungal communities structure) for all 12 composite samples (Figure 2). Among the three variables, it was observed that plant components (rhizosphere soil and roots) significantly influenced bacterial and fungal community composition. The separation of samples at the one axis for bacterial community composition indicates that the influence of this variable on bacterial community structure was more significant than on fungal community structure. Sample types and locations have little impact on bacterial and fungal communities. A different pattern was observed using PCoA, with a difference of 37.17% and 18.67% in bacterial and fungal community composition, respectively. Distance heatmap graphs based on Weighted UniFrac (based on abundances of taxa) and Unweighted UniFrac (sensitive to rare taxa) of all 12 samples were generated to estimate the beta diversity of bacterial and fungal communities (Supplementary Figure 3).

### Effects of Different Locations, Plant Components, and Sample Types on Alpha Diversity

**FIGURE 3:**
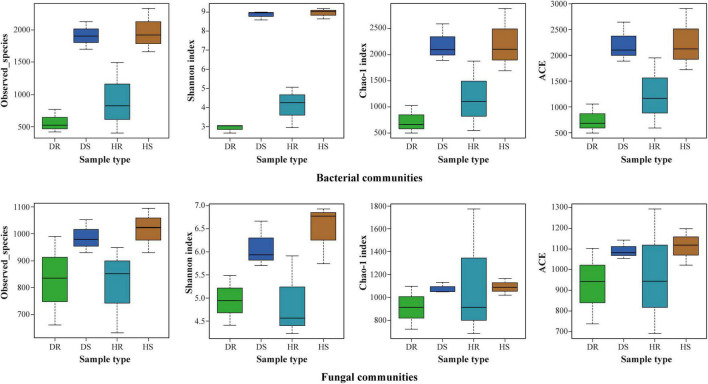
Boxplot of bacterial **(top)** and fungal **(bottom)** showing alpha diversity indexes of flue-cured tobacco plants under three variables. HS, healthy rhizosphere soil; DS, diseased rhizosphere soil; HR, healthy roots; DR, diseased roots.

The observed species, Shannon, Chao 1, and ACE at cutoff levels of 3% are shown in Figure 3. The locations have little impact on alpha diversity indices of bacterial communities compared with fungal communities (Supplementary Table 2). In plant components (rhizosphere soil and roots), alpha diversity indices of observed species, Shannon, Chao 1, and ACE for rhizosphere soil were found higher than those of roots for both bacterial and fungal communities. It indicates that the number of bacteria and fungi in the rhizosphere was higher than in roots. Among the sample types (healthy and diseased), alpha diversity indices of bacterial and fungal communities were more elevated in healthy samples than in diseased samples.

### Analysis of Operational Taxonomy Units

**FIGURE 4:**
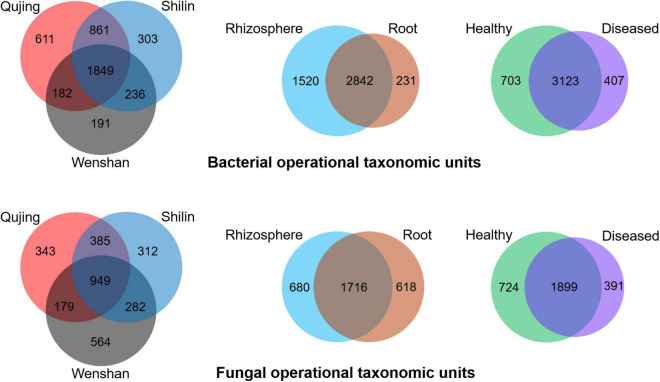
Distribution of bacterial **(top)** and fungal **(bottom)** operational taxonomic units in three variables, i.e., locations (Qujing, Shilin, and Wenshan), plant components (rhizosphere soil and roots), and sample types (healthy and diseased).

Operational taxonomic units count was recorded maximum in rhizosphere soil samples than in root samples. For bacterial and fungal communities, the rhizosphere soil exhibited a high diversity and richness in OTUs compared with roots, and high diversity was found in healthy plant samples than in diseased plant samples (Figure 4). Analysis of OTUs revealed that a total of 4,233 and 3,014 specific OTUs were recovered for both bacterial and fungal communities from different locations (Qujing, Shilin, and Wenshan), respectively, and 1,849 (bacterial) and 949 (fungal) OTUs were found as common OTUs. Plant components (rhizosphere soil and roots) significantly impact the bacterial communities than fungal communities, and fungal OTU count in rhizosphere soil and roots was almost the same. In plant components, the specific bacterial OTUs in rhizosphere soil (1,520) were significantly higher than the specific OTUs (231) in the root tissue, and 2,482 OTUs were common. The specific fungal OTUs in root tissues (680) were slightly higher than specific OTUs (618) in rhizosphere soil, and 1,716 common OTUs were found in rhizosphere soil and roots. Further analysis of OTUs among sample types (healthy and diseased) showed that a total of 703 and 407 unique bacterial OTUs and 3,123 common bacterial OTUs were found among healthy and diseased plants, respectively. The unique fungal OTUs in healthy samples (724) were significantly higher than diseased samples (391), and 1,899 OTUs were found as common. The results indicate that bacterial wilt pathogen had a more significant impact on bacterial communities than fungal communities.

### Bacterial and Fungal Community Composition at Phylum Level

**FIGURE 5:**
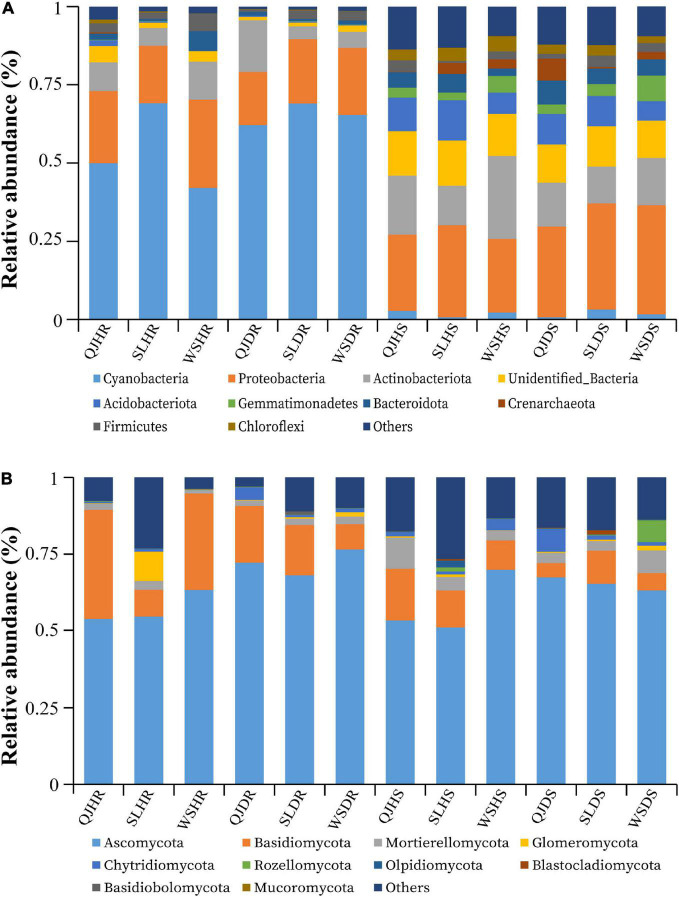
Relative abundance bar plots at phylum level based on the species annotation results in 12 composite samples (average of three replicates per sample) of flue-cured tobacco plants under three variables. **(A)** Relative abundance at the phylum level in bacterial communities and **(B)** relative abundance at the phylum level in fungal communities. QJ, Qujing; SL, Shilin; WS, Wenshan; HS, healthy rhizosphere soil; DS, diseased rhizosphere soil; HR, healthy roots; DR, diseased roots.

The top 10 bacterial and fungal phyla with relative abundance greater than 1% are shown in Figure 5 and Supplementary Table 3. The dominant bacterial phyla in all rhizosphere soil and root samples with a relative abundance greater than 1% are Acidobacteriota, Actinobacteriota, Bacteroidota, Cyanobacteria, Crenarchaeota, Chloroflexi, Gemmatimonadetes, Firmicutes, Proteobacteria, Unidentified_Bacteria, and others (Figure 5A). The dominant fungal phyla in all rhizosphere soil and root samples with a relative abundance greater than 1% are Ascomycota, Basidiomycota, Blastocladiomycota, Basidiobolomycota, Chytridiomycota, Glomeromycota, Mortierellomycota, Mucoromycota, Rozellomycota, Olpidiomycota, and others (Figure 5B). Among the three variables (locations, plant components, and sample types), plant components (rhizosphere soil and roots) significantly influence the bacterial community composition. High relative abundance of phyla Cyanobacteria (average of 59.61%) and Proteobacteria (average of 21.44%) was found in roots; however, phyla Proteobacteria (average of 29.20%) and Actinobacteria (average of 16.46%) were found in high relative abundance in rhizosphere soil (Figure 5A). No significant impact was observed of these three variables on fungal community composition. Fungal phyla such as Ascomycota (average of 65 and 20%) and Basidiomycota (average of 62 and 10%) were present in high relative abundance both in root and rhizosphere soil samples, respectively. However, phylum Basidiomycota was found in low relative abundance in rhizosphere soil (average 10%) than in roots (average 20%) (Figure 5B).

### Relative Abundance of Bacterial and Fungal Community Composition at Genera Level

**FIGURE 6:**
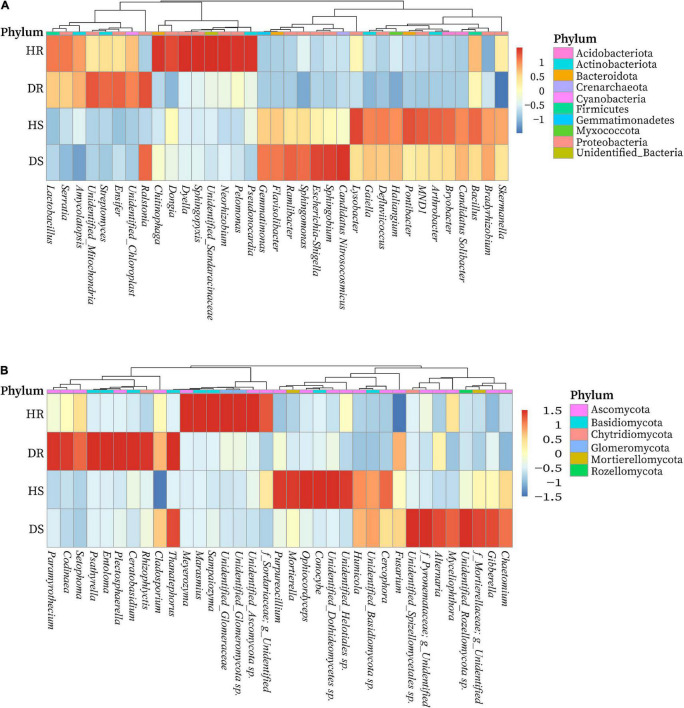
Relative abundance heatmaps at the genus level for top 35 bacterial **(A)** and fungal **(B)** genera in group-wise comparison under three variables. HS, healthy rhizosphere soil; DS, diseased rhizosphere soil; HR, healthy roots; DS, diseased roots.

We determined the relative abundance of the top 35 bacterial and fungal genera in the above screened top 10 bacterial and fungal phyla. On the basis of the species relative abundance in all samples, the top 35 bacterial and fungal genera were selected to create a heatmap to determine which genera present in high or low abundance in respective sample type (healthy and diseased). The relative abundance heatmaps of the top 35 bacterial and fungal genera in group-wise comparison under three variables (locations, plant components, and sample types) are shown in Figure 6. Bacterial genera such as *Streptomyces*, *Amycolatopsis*, and *Ensifer*; *Chitinophaga*, *Dyella*, Dongia, *Neorhizobium*, *Pelomonas*, *Pseudonocardia*, and *Sphingopyxis*; *Esherichia*-*Shigella*, *Sphingomonas*, *Ramlibacter*, *Flavisobater*, *Gemmatimonas*, and *Sphingobium*; and *Lysobacter*, *Arthrobacter*, *Bryobacter*, *Bacillus*, *MND1*, *Gaiella*, and *Pontibacter* were found in high relative abundance in diseased roots (DR), healthy roots (HR), diseased rhizosphere soil (DS), and healthy rhizosphere soil (HS), respectively. However, a high abundance of genus *Ralstonia* was present in diseased rhizosphere soil and root samples (Figure 6A). Similarly, fungal genera such as *Chaetomium*, *Gibberella*, *Myceliophthora*, and *Alternaria*; *Ophiocordyceps*, *Conocybe*, *Cercophora*, *Purpureocillium*, *Humicola*, and *Mortierella*; *Codinaea*, *Rhizophlycits*, *Ceratobasidium*, *Entoloma*, *Paramyrothecium*, *Setophoma*, and *Psathyrella*; and *Marasmius*, *Meyerozyma*, *Sampaiozyma*, and Unidentified_Ascomycota sp. were found in high relative abundance in DS, HS, DR, and HR, respectively. Whereas genus *Thanatephorus* was highly abundant with diseased rhizosphere soil and root samples (Figure 6B).

### Relative Abundance of Bacterial and Fungal Communities at the Species Level

**FIGURE 7:**
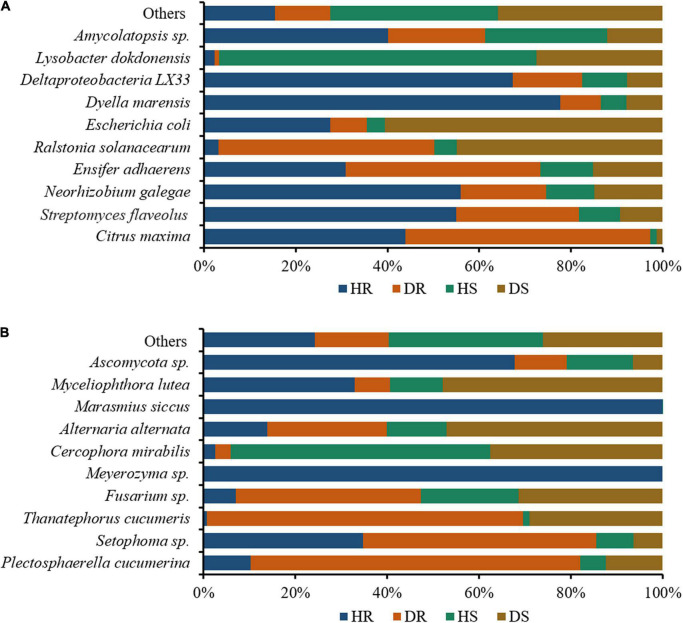
Relative abundance bar plots for top 10 bacterial **(A)** and fungal **(B)** species in group-wise comparison under three variables. HS, healthy rhizosphere soil; DS, diseased rhizosphere soil; HR, healthy roots; DS, diseased roots.

We assessed the relative abundance of the top 10 bacteria and fungi in the top 35 bacterial and fungal genera. The relative abundance bar plots for the top 10 bacteria and fungi in group-wise comparison under three variables (locations, plant components, and sample types) are shown in Figure 7. A high abundance of bacterial wilt pathogen *Ralstonia solanacearum* was found in diseased roots and rhizosphere soil. *Neorhizobium galegae* (well-known nitrogen fixer), *Ensifer adhaerens* (a bacterial predator of bacteria in the soil), and *Lysobacter dokdonensis* were found in high abundance in HR, DR, and HS, respectively (Figure 7A). Fungal species such as *Alternaria alternata* and *Thanatephorus cucumeris*, disease causing agents of tobacco brown spot and leaf spot disease, respectively, were found in high abundance with diseased rhizosphere soil and root samples. Moreover, higher abundance of *Fusarium* sp. was found in DR and DS (Figure 7B).

### Characteristics of Co-occurrence Network

**FIGURE 8:**
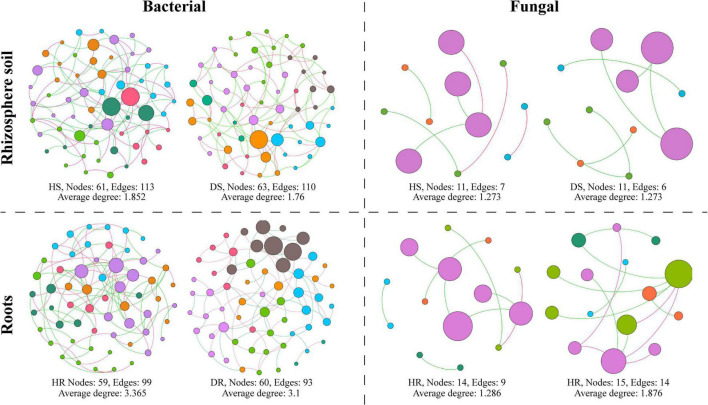
Co-occurrence network analysis of bacterial and fungal communities associated with healthy and diseased samples collected from different plant components (rhizosphere soil and roots). HS, healthy soil; DS, diseased soil; HR, healthy roots; DR, diseased roots.

A microbial co-occurrence network was constructed for bacterial and fungal OTUs at the phylum level for each healthy and diseased sample associated with different plant components (rhizosphere soil and roots) (Figure 8). It was observed that, for bacterial communities, the average degree, number of nodes, and number of edges were found higher in healthy samples compared with diseased samples. In the fungal communities, the average degree, number of nodes, and number of edges were found higher in diseased samples than healthy samples while have no effect on the rhizosphere network. It indicates that the connection degree between members of the bacterial microbial network increased in healthy samples and members interact, whereas the fungal microbial network showed an opposite trend.

## Discussion

*Ralstonia solanacearum* is a serious threat to flue-cured tobacco production worldwide, including China. It is estimated that approximately 47,133 hm^–2^ lands are affected by this pathogen, resulting in millions dollars losses every year (Cai et al., 2021). Many integrated disease management strategies are adopted to control the incidence of bacterial wilt pathogen (Jiang et al., 2017). In recent years, biological control *via* disease suppressive biocontrol agents is considered a promising approach to reduce the incidence of many field crop diseases (Munir et al., 2021; Ahmed et al., 2022). Studies have proven that soil health and rhizosphere microbial diversity are the key factors responsible for soilborne disease occurrence and plant health (Köberl et al., 2013; Dong et al., 2018; Hao and Ashley, 2021).

Soil sickness affects both crop quality and yields when the same crop or its relative species are continuously grown in the same soil under a monocropping system (Zhang et al., 2020b). The rhizosphere microbiome acts as the first line of defense against soilborne pathogen infection and abiotic stress (Bulgarelli et al., 2013; Mendes et al., 2013). In previous studies, flue-cured tobacco bacterial wilt diseases associated with bacterial and fungal communities were studied by direct isolation and high-throughput sequencing technique. However, most studies performed on rhizosphere microbial diversity aimed at bacterial communities with little knowledge about fungal communities of flue-cured tobacco grown on a specific location in pots and field experiments after artificial inoculation of *R. solanacearum* combined with other biocontrol agents.

In this study, we provided a comprehensive view of the naturally occurring core microbiome of healthy and bacterial wilt–infected (diseased) flue-cured tobacco plants. We presented an in-depth appraisal of bacterial and fungal communities associated with different locations, sample types, and plant components through 16S and ITS1-5f high-throughput sequencing analysis. Our results support the concept that distinct plant components have a significant impact on bacterial and fungal communities, irrespective of sample types and location.

Different plant components have been observed to study the impact of rhizospheric and endophytic bacterial communities on the growth and health of plants with no specific assemblage pattern (Compant et al., 2008; Lundberg et al., 2012; Lebeis et al., 2015). The plant’s rhizosphere acts as the primary host, hot spot habitat, and passage for colonization of microbes into different plant components through roots, especially for bacterial communities (Bulgarelli et al., 2013; Kaushal et al., 2020). In our study, samples were collected from fields where flue-cured tobacco has been continuously grown for the past 10 years as an annual crop for commercial production. Therefore, infected soil and tools used for mechanical operations are primary carriers of bacterial wilt pathogen from one field to another.

Our study demonstrated that rhizosphere soil greatly influences microbial diversity and host more bacterial and fungal communities than roots. Most of the communities present in roots are also present in rhizosphere soil in all locations. The results suggest the microbial communities present in roots were transferred and colonized from the rhizosphere soil. Studies have proven that host plants selectively promote colonization of specific bacterial and fungal communities in roots from rhizosphere soil (Turnbaugh et al., 2007; Lundberg et al., 2012). It was found that rhizosphere soil showed a high microbial diversity than roots. However, healthy tobacco plant rhizosphere soil and root samples host a high diversity of bacterial and fungal communities than bacterial wilt–infected tobacco plant samples. This may be due to disease stress, which is directly linked to the decreased value of available carbon for microbes in the rhizosphere and regulating specific microbe’s growth (Kaushal et al., 2020). Flue-cured tobacco plant resistance and severity of *R. solanacearum* in different plant components also contribute to the prevalence of microbial communities. Bacterial and fungal OTU count was recorded maximum in all locations and plant components in rhizosphere soil samples than in the root samples.

Host plant environment is known to support or suppress the colonization of certain bacterial and fungal genera with different plant components. Thus, bacterial and fungal communities in these specific plant components are either boosted or exhausted (Kaushal et al., 2020). We observed the core stable bacterial and fungal communities in all sample types that were colonized in different plant components with respect to OTUs distribution. In all rhizosphere soil and root samples for bacterial community composition phylum, Proteobacteria and Cyanobacteria were found in high relative abundance, respectively. Whereas for fungal community composition, phyla Ascomycota and Basidiomycota were found in high relative abundance in both rhizosphere soil and root samples. Results showed that bacterial wilt pathogen has a great impact and significantly influences the bacterial communities compared with fungal communities.

Most of the diverse bacteria and fungi can enhance plant growth, suppress disease incidence, and confer other biological functions that benefit the plants (Santoyo et al., 2016; Gouda et al., 2018). A fraction of core bacterial communities identified in this study are known as plant growth promoters, enhance resistance in plants, and suppress the incidence of disease *via* production of a specific antibiotic, volatile organic compounds, secondary metabolites, and nitrogen fixation in many fields crops. Bacterial genera such as *Amycolatopsis*, *Bacillus*, *Streptomyces*, *Ensifer*, *Sphingopyxis*, *Neorhizobium*, *Pseudonocardia*, *Sphingomonas*, *Haliangium*, *Bradyrhizobium*, *Arthrobacter*, *Bryobacter*, and *Lysobacter* were found in high relative abundance, which contribute to plant growth promotion, nutrition acquisition, suppression of soilborne diseases and other plant pathogens. However, a minor fraction of fungal communities such as *Gibberella* and *Purpureocillium* was found as plant growth promoters and biocontrol agents, whereas others are saprophytic and pathogenic and cause disease in many important field crops.

Bacterial wilt pathogen *R. solanacearum* was highly abundant in diseased rhizosphere soil and root. A high abundance of the bacterium was found with nitrogen-fixing ability, producing secondary metabolites, and bacterial predator of bacteria in the soil such as *Neorhizobium galegae*, *Lysobacter dokdonensis*, and *Ensifer adhaerens*. Plants pathogenic fungi such as *Plectosphaerella cucumerina*, *Alternaria alternata*, *Thanatephorus cucumeris*, and *Fusarium* sp. were found in high relative abundance diseased rhizosphere soil and root. Thus, we conclude that these beneficial bacterial communities are positively related to flue-cured tobacco plant health, whereas the pathogenic fungal communities enhance the population of *R. solanacearum* and the incidence of bacterial wilt disease. Our results are in accordance with the study of Jiang et al. (2017), who reported that the incidence of tobacco bacterial wilt disease increases when it prevails with other root rot pathogen *P. nicotiana*. Analysis of co-occurrence networks showed that *R. solanacearum* has a significant impact on the bacterial community structure compared with fungal communities. Fewer negative correlations were found between bacterial communities in healthy plants compared to diseased plants.

## Conclusion

In this study, we conclude that flue-cured tobacco plants host a range of bacterial and fungal communities. Plant components significantly impact bacterial and fungal communities. Rhizosphere soil is enriched with bacterial and fungal communities compared to roots, whereas high diversity was found in healthy plants rather than diseased plants. By comparing the microbial communities with other crops, we suggest that several bacterial communities discovered in the flue-cured tobacco microbiome aid in plant growth promotion and disease suppression. A fraction of fungal communities pathogenic to flue-cured tobacco was found in diseased plants, which correlates with the occurrence of tobacco bacterial wilt disease. However, future work should focus on studying the microbiome of flue-cured tobacco cultivars with resistance to bacterial wilt pathogen both as naturally occurring and artificial inoculation. This will provide experimental and theoretical information, that microbiome of flue-cured tobacco can be engineered to manage the bacterial wilt disease for better yield and quality production.

## Data Availability Statement

The datasets presented in this study can be found in online repositories. The names of the repository/repositories and accession number(s) can be found in the article/Supplementary Material.

## Author Contributions

ZZ and GJ designed the experiment. WA, QL, SL, and JZ visited the tobacco-growing areas and collected samples. WA, ZD, and JY analyzed data and drew figures. WA, SM, and SK wrote the initial draft of manuscript. WA, SM, GJ, and ZZ wrote, reviewed, and edited final draft of the manuscript. All authors contributed to the final draft of the manuscript and agreed to the published version of the manuscript.

## Conflict of Interest

The authors declare that the research was conducted in the absence of any commercial or financial relationships that could be construed as a potential conflict of interest.

## Publisher’s Note

All claims expressed in this article are solely those of the authors and do not necessarily represent those of their affiliated organizations, or those of the publisher, the editors and the reviewers. Any product that may be evaluated in this article, or claim that may be made by its manufacturer, is not guaranteed or endorsed by the publisher.
