# Supplementary material for: Microbial Cross-Talk: Dissecting the Core Microbiota Associated With Flue-Cured Tobacco (Nicotiana tabacum) Plants Under Healthy and Diseased State
Source: Front Microbiol. 2022 Apr 14;13:845310. doi: 10.3389/fmicb.2022.845310 (PMC9048796; doi:10.3389/fmicb.2022.845310)
Supplement: Supplementary file 1 [file Data_Sheet_1.docx]

**Microbial Cross-Talk: Dissecting the core microbiota associated with flue-cured tobacco (*Nicotiana tabacum*) plants under healthy and diseased state**

Waqar Ahmed^1,2,3^, Zhenlin Dai^2,3^, Qi Liu^2,3^, Shahzad Munir^2^, Jun Yang^2,3,4^, Samantha C. Karunarathna^5^, Shichen Li^6^, Jinhao Zhang^2,3^, Guanghai Ji^2,3,^* and Zhengxiong Zhao^1,^*

^1^College of Resources and Environment, Yunnan Agricultural University, Kunming, 650201, Yunnan, China

^2^State Key Laboratory for Conservation and Utilization of Bio-resources in Yunnan, Yunnan Agricultural University, Kunming 650201, Yunnan, China

^3^Key Laboratory of Agro-Biodiversity and Pest Management of Ministry of Education, Yunnan Agricultural University, Kunming 650201, China

^4^College of Resources, Environment, and Chemistry, Chuxiong Normal University, Chuxiong 675000, China

^5^Biological Resources Protection and Utilization, College of Biological Resources and Food Engineering, Qujing Normal University, Qujing 655011, China

^6^College of Agronomy and Biotechnology, Yunnan Agricultural University, Kunming, 650201, Yunnan, China

***Correspondence authors**

Zhengxiong Zhao

E-mail: [zhaozx0801@163.com](mailto:zhaozx0801@163.com)

Guanghai Ji

E-mail: [jghai001@163.com](mailto:jghai001@163.com)

| **Table S1; Data obtained from sequencing data of V3-V4 and ITS1-5f variable regions of 16S and ITS rRNA of bacteria and fungi, respectively.** | | | | | |
| --- | --- | --- | --- | --- | --- |
| **Sample Id** | **Raw reads (#)** | **Clean reads (#)** | **Average (bP)** | **Q20 (%)** | **Q30 (%)** |
| **Bacteria (16S; V3-V4)** | | | | | |
| QJDS | 66,441±51.389 | 59,595±25.935 | 414±1.247 | 97.08 | 91.66 |
| QJHS | 97,416±64.499 | 93,172±62.034 | 417±1.059 | 98.62 | 95.4 |
| QJHR | 103,253±218.802 | 99,669±44.145 | 410±1.868 | 98.62 | 95.4 |
| QJDR | 98,391±103.789 | 86,134±81.270 | 407±1.032 | 97.39 | 92.4 |
| SLHS | 61,429±85.947 | 56,086±78.845 | 415±1.159 | 97.14 | 91.81 |
| SLDS | 92,886±37.857 | 88,823±15.629 | 416±1.342 | 98.58 | 95.31 |
| SLHR | 103,577±119.973 | 91,345±46.952 | 408±1.013 | 97.35 | 92.24 |
| SLDR | 99,641±377.143 | 88,183±17.115 | 408±1.215 | 97.35 | 92.31 |
| WSHS | 58,259±313.563 | 53,547±101.535 | 413±1.287 | 96.98 | 91.32 |
| WSDS | 72,223±96.389 | 63,767±69.653 | 415±1.196 | 96.87 | 91.13 |
| WSHR | 108,739±188.669 | 91,859±37.361 | 410±1.632 | 97.16 | 91.92 |
| WSDR | 108,202±124.991 | 93,542±96.579 | 409±1.022 | 97.29 | 92.14 |
| **Fungi (ITS; 1-5f)** | | | | | |
| QJDS | 78,053±78.256 | 72,775± 39.458 | 243± 3.77 | 99.42 | 98.1 |
| QJHS | 99,520±109.648 | 93,076± 84.632 | 245± 2.65 | 99.22 | 97.6 |
| QJHR | 95,126±96.235 | 91,726± 69.148 | 257± 3.46 | 98.97 | 96.97 |
| QJDR | 98,472±101.521 | 96,361± 78.615 | 246± 2.51 | 99.1 | 97.04 |
| SLHS | 111,472±135.653 | 100,079± 96.568 | 249± 2.32 | 99.08 | 97.06 |
| SLDS | 109,058±115.478 | 101,502± 76.658 | 233± 1.963 | 99.37 | 98.11 |
| SLHR | 97,427±92.692 | 92,338± 63.425 | 237± 2.012 | 98.95 | 97.07 |
| SLDR | 99,962±98.456 | 95,260± 4.963 | 236± 1.984 | 99.34 | 97.99 |
| WSHS | 108,443±125.694 | 103,893± 85.785 | 243± 2.57 | 99.28 | 97.69 |
| WSDS | 92,356±84.567 | 85,860± 56.632 | 239± 2.26 | 99.46 | 98.17 |
| WSHR | 87,219±76.129 | 81,282± 39.856 | 235± 1.846 | 99.35 | 98.12 |
| WSDR | 101,050±105.631 | 98,066± 72.639 | 263± 3.84 | 98.76 | 95.57 |
| Here; Q20 and Q30 are the percentages of bases with base quality values greater than 20 (sequencing error rate less than 1%) and 30 (sequencing error rate less than 0.1%). Data is represented as standard error of means (±SEM, n=3). | | | | | |

| **Table S2; Alpha diversity analysis indexes (shannon, simpson, chao1, ACE, goods coverage, PD whole tree) for 16S rRNA and ITS gene libraries from different samples at the 97% consistency threshold level (n=3, ±SEM).** | | | | | | |
| --- | --- | --- | --- | --- | --- | --- |
| **Sample Id** | **Observed species** | **Shannon** | **Simpson** | **Chao1** | **ACE** | **PD whole tree** |
| **Bacteria (16S; V3-V4)** | | | | | | |
| QJDS | 1903±24.553 | 8.999±0.010 | 0.994±0.0016 | 2096.28±45.729 | 2107.68±43.124 | 142.791±0.720 |
| QJHS | 2329±36.786 | 9.176±0.015 | 0.993±0.0012 | 2881.415±49.684 | 2911.619± | 183.16±1.125 |
| QJHR | 1497±19.568 | 5.063±0.003 | 0.75±0.0011 | 1877.96±32.487 | 1956.641± | 143.721±1.014 |
| QJDR | 423±14.236 | 3.048±0.001 | 0.611±0.0010 | 499±10.265 | 498.374± | 52.556±0.362 |
| SLHS | 1920±23.689 | 9.011±0.017 | 0.994±0.0012 | 2101.958±56.325 | 2124.924± | 150.213±0.894 |
| SLDS | 2124±31.456 | 8.958±0.011 | 0.994±0.0013 | 2586.885±62.542 | 2646.764± | 173.975±1.692 |
| SLHR | 827±15.656 | 2.947±0.001 | 0.526±0.0010 | 1096.948±26.314 | 1173.026± | 86.891±0.986 |
| SLDR | 769±12.598 | 2.673±0.001 | 0.518±0.0010 | 1023.159±19.412 | 1055.301± | 97.149±1.001 |
| WSHS | 1660±18.248 | 8.641±0.019 | 0.99±0.0014 | 1686.553±15.214 | 1722.85± | 141.167±1.631 |
| WSDS | 1702±22.785 | 8.587±0.016 | 0.991±0.0026 | 1885.023±21.459 | 1891.461± | 136.352±1.572 |
| WSHR | 408±10.587 | 4.261±0.001 | 0.813±0.0019 | 547.688±12.215 | 594.77± | 72.39±0.465 |
| WSDR | 526±15.456 | 3.064±0.001 | 0.575±0.001 | 663.811±14.569 | 684.899±5.316 | 63.326±0.541 |
| **Fungi (ITS; 1-5f)** | | | | | | |
| QJDS | 930±14.621 | 5.707±0.0056 | 0.943±0.0011 | 1045.831±22.314 | 1054.842±25.364 | 355.174±2.651 |
| QJHS | 1023±18.652 | 5.743±0.0062 | 0.934±0.0014 | 1088.707±19.635 | 1118.764±33.452 | 361.566±1.986 |
| QJHR | 851±13.259 | 4.234±0.0032 | 0.839±0.0016 | 914.268±20.214 | 943.951±14.125 | 349.762±0.942 |
| QJDR | 661±9.647 | 4.413±0.0034 | 0.891±0.0014 | 721.024±16.364 | 738.454±10.254 | 248.145±0.541 |
| SLHS | 1095±10.235 | 6.928±0.0165 | 0.981±0.01452 | 1166.968±26.314 | 1197.147±12.498 | 415.495±0.864 |
| SLDS | 979±8.694 | 5.939±0.0101 | 0.94±0.0163 | 1056.037±20.365 | 1081.244±15.789 | 330.993±0.569 |
| SLHR | 949±9.963 | 5.914±0.0158 | 0.953±0.0012 | 1773.053±25.147 | 1292.587±29.641 | 411.296±0.762 |
| SLDR | 835±6.314 | 4.942±0.0026 | 0.837±0.0069 | 914.067±13.364 | 941.417±9.621 | 312.836±0.964 |
| WSHS | 930±12.364 | 6.771±0.0354 | 0.975±0.0196 | 1019.425±10.415 | 1021.296±11.985 | 266.852±0.235 |
| WSDS | 1053±11.426 | 6.665±0.045 | 0.971±0.0075 | 1131.147±16.398 | 1142.25±10.548 | 327.493±1.235 |
| WSHR | 632±5.234 | 4.568±0.0012 | 0.894±0.0084 | 685.308±6.694 | 691.735±5.235 | 303.206±1.012 |
| WSDR | 990±10.647 | 5.491±0.0654 | 0.858±0.0013 | 1097.201±9.6478 | 1102.865±15.489 | 396.147±2.983 |

| **Table S3; Relative abundance of top 10 dominant bacterial and fungal phyla in different sample.** | | | | | | | | | | | | |
| --- | --- | --- | --- | --- | --- | --- | --- | --- | --- | --- | --- | --- |
| **Bacterial** | | | | | | | | | | | | |
| **Phyla** | **QJHR** | **SLHR** | **WSHR** | **QJDR** | **SLDR** | **WSDR** | **QJHS** | **SLHS** | **WSHS** | **QJDS** | **SLDS** | **WSDS** |
| Cyanobacteria | 0.5001±0.084 | 0.6913±0.105 | 0.4205±0.051 | 0.6211±0.065 | 0.6901±0.086 | 0.6535±0.026 | 0.0269±0.004 | 0.0067±0.002 | 0.0224±0.015 | 0.0072±0.003 | 0.0319±0.016 | 0.0165±0.015 |
| Proteobacteria | 0.2298±0.007 | 0.1837±  0.098 | 0.2819±0.063 | 0.1701±0.029 | 0.2056±0.054 | 0.2148±0.063 | 0.2444±0.069 | 0.2944±0.075 | 0.2354±0.009 | 0.2896±0.056 | 0.3394±0.098 | 0.3487±0.066 |
| Actinobacteriota | 0.0921±0.005 | 0.0562±0.024 | 0.1215±0.049 | 0.1652±0.05 | 0.0409±0.007 | 0.0509±0.031 | 0.1878±0.003 | 0.1257±0.010 | 0.2646±0.018 | 0.1405±0.029 | 0.1177±0.074 | 0.1512±0.029 |
| Unidentified_Bacteria | 0.0519±0.010 | 0.0170±0.018 | 0.0338±0.005 | 0.0110±0.012 | 0.0123±0.007 | 0.0205±0.031 | 0.1423±0.003 | 0.1452±0.016 | 0.1344±0.004 | 0.1224±0.016 | 0.1288±0.005 | 0.1189±0.015 |
| Acidobacteriota | 0.0181±0.005 | 0.0045±0.002 | 0.0003±0.102 | 0.0026±0.015 | 0.0031±0.002 | 0.0018±0.001 | 0.1071±0.104 | 0.1283±0.001 | 0.0681±0.004 | 0.0968±0.001 | 0.0960±0.005 | 0.0623±0.029 |
| Gemmatimonadetes | 0.0018±0.001 | 0.0008±0.003 | 0.00±0.00 | 0.00±0.00 | 0.0012±0.001 | 0.0007±0.003 | 0.0322±0.004 | 0.0244±0.016 | 0.0530±0.010 | 0.0301±0.018 | 0.0384±0.004 | 0.0815±0.102 |
| Bacteroidota | 0.0196±0.006 | 0.0068±0.009 | 0.0638±0.043 | 0.0125±0.031 | 0.0069±0.001 | 0.0133±0.012 | 0.0499±0.0214 | 0.0597±0.010 | 0.0247±0.016 | 0.0770±0.0178 | 0.0504±0.025 | 0.0517±0.036 |
| Crenarchaeota | 0.0036±0.002 | 0.0001±0.001 | 0.00±0.00 | 0.0002±0.001 | 0.0001±0.001 | 0.0005±0.003 | 0.0006±0.001 | 0.0345±0.004 | 0.0285±0.018 | 0.0693±0.031 | 0.0035±0.016 | 0.0243±0.004 |
| Firmicutes | 0.0288±0.029 | 0.0206±0.012 | 0.0554±0.005 | 0.0072±0.001 | 0.0275±0.016 | 0.0285±0.004 | 0.0366±0.016 | 0.0063±0.003 | 0.0253±0.018 | 0.0159±0.074 | 0.0369±0.018 | 0.0277±0.004 |
| Chloroflexi | 0.0116±0.015 | 0.0033±0.003 | 0.0004±0.001 | 0.0017±0.010 | 0.0019±0.001 | 0.0012±0.001 | 0.0351±0.015 | 0.0432±0.003 | 0.0486±0.016 | 0.0294±0.0152 | 0.0333±0.018 | 0.0214±0.016 |
| Others | 0.0420±0.007 | 0.0153±0.074 | 0.0222±0.102 | 0.0080±0.003 | 0.0098±0.102 | 0.0138±0.003 | 0.1367±0.098 | 0.1312±0.029 | 0.0945±0.024 | 0.1213±0.074 | 0.1232±0.066 | 0.0952±0.031 |
| **Fungal** | | | | | | | | | | | | |
| Ascomycota | 0.5386±0.094 | 0.5471±0.116 | 0.6335±0.149 | 0.7226±0.022 | 0.6815±0.054 | 0.7651±0.033 | 0.5346±0.095 | 0.5110±0.119 | 0.6991±0.159 | 0.6748±0.195 | 0.6538±0.183 | 0.6315±0.159 |
| Basidiomycota | 0.3556±0.061 | 0.0869±0.033 | 0.3136±0.058 | 0.1842±0.083 | 0.1633±0.169 | 0.0825±0.022 | 0.1676±0.017 | 0.1204±0.005 | 0.0962±0.064 | 0.0465±0.01 | 0.1075±0.005 | 0.0570±0.022 |
| Mortierellomycota | 0.0189±0.02 | 0.0285±0.023 | 0.0114±0.003 | 0.0178±0.029 | 0.0201±0.035 | 0.0246±0.021 | 0.1016±0.101 | 0.0444±0.009 | 0.0312±0.002 | 0.0333±0.039 | 0.0308±0.024 | 0.0737±0.064 |
| Glomeromycota | 0.0027±0.001 | 0.0951±0.004 | 0.0005±0.001 | 0.0013±0.010 | 0.0051±0.004 | 0.0143±0.003 | 0.0045±0.021 | 0.0079±0.032 | 0.0010±0.001 | 0.0026±0.004 | 0.0048±0.023 | 0.0153±0.015 |
| Chytridiomycota | 0.0049±0.001 | 0.0090±0.003 | 0.0019±0.001 | 0.0420±0.038 | 0.0073±0.004 | 0.0114±0.013 | 0.0135±0.020 | 0.0094±0.001 | 0.0370±0.024 | 0.0756±0.064 | 0.0136±0.1639 | 0.0119±0.013 |
| Rozellomycota | 0.0016±0.02 | 0.0005±0.001 | 0.0008±0.002 | 0.0015±0.0120 | 0.0006±0.001 | 0.0015±0.010 | 0.0006±0.001 | 0.0136±0.020 | 0.0012±0.008 | 0.0007±0.001 | 0.0021±0.010 | 0.0705±0.070507 |
| Olpidiomycota | 0.0006±0.001 | 0.0016±0.013 | 0.00±0.00 | 0.0002±0.001 | 0.00±0.00 | 0.0001±0.00 | 0.0020±0.001 | 0.0222±0.035 | 0.00±0.00 | 0.0008±0.001 | 0.0014±0.001 | 0.0021±0.001 |
| Blastocladiomycota | 0.0001±0.001 | 0.0004±0.001 | 0.0006±0.001 | 0.00±0.00 | 0.00±0.00 | 0.0001±0.001 | 0.0007±0.002 | 0.0050±0.0120 | 0.00±0.00 | 0.0009±0.003 | 0.0137±0.010 | 0.00±0.00 |
| Basidiobolomycota | 0.00±0.00 | 0.00±0.00 | 0.00±0.00 | 0.0001±0.001 | 0.0105±0.001 | 0.0007±0.003 | 0.00±0.00 | 0.0001±0.001 | 0.0004±0.001 | 0.00±0.00 | 0.00±0.00 | 0.0005±0.001 |
| Mucoromycota | 0.00±0.00 | 0.00±0.00 | 0.00±0.00 | 0.0002±0.001 | 0.00±0.00 | 0.00±0.00 | 0.0001±0.001 | 0.00±0.00 | 0.00±0.00 | 0.0006±0.002 | 0.00±0.00 | 0.0002±0.001 |
| Others | 0.0764±0.015 | 0.2301±0.063 | 0.0371±0.004 | 0.0296±0.0124 | 0.1109±0.05 | 0.0993±0.024 | 0.1744±0.017 | 0.2655±0.063 | 0.1335±0.066 | 0.1637±0.029 | 0.1717±0.074 | 0.13689±0.019 |
| Note: Relative abundance at phylum level in bacterial communities of the top 10 species with highest abundance at each classification level (Phylum, Class, Order, Family, Genus). Data is represented as standard error of means (±SEM, n=3). | | | | | | | | | | | | |


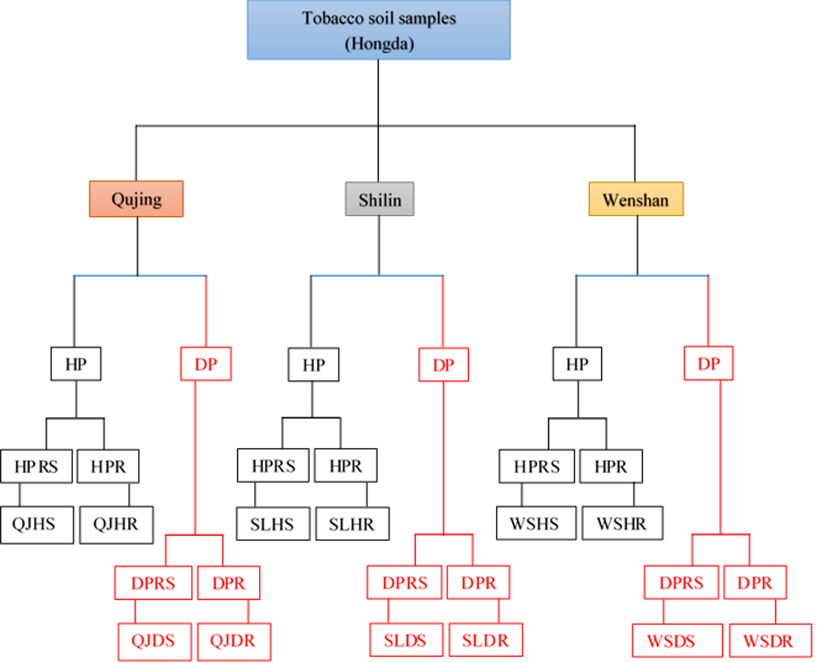


**Figure S1:** Overview of sampling strategy from healthy and bacterial wilt infected diseased flue-cured tobacco (Hongda) plants. Here: Qujing; QJ, Shilin; SL, Wenshan; WS, HP; healthy plants, DP; diseased plants, HPRS; Healthy plants rhizosphere soil, HPR; Healthy plants roots, DPRS; Diseased plants rhizosphere soil, DPR; Diseased plants roots, QJHS; Qujing healthy plants rhizosphere soil, QJHR; Qujing healthy plants roots, QJDS; Qujing diseased plants rhizosphere soil, QJDR; Qujing diseased plants roots, SLHS; Shilin healthy plants rhizosphere soil, SLHR; Shilin healthy plants roots, SLDS; Shilin diseased plants rhizosphere soil, SLDR; Shilin diseased plants roots, WSHS; Wenshan healthy plants rhizosphere soil, WSHR; Wenshan healthy plants roots, WSDS; Wenshan diseased plants rhizosphere soil, WSDR; Wenshan diseased plants roots.

**
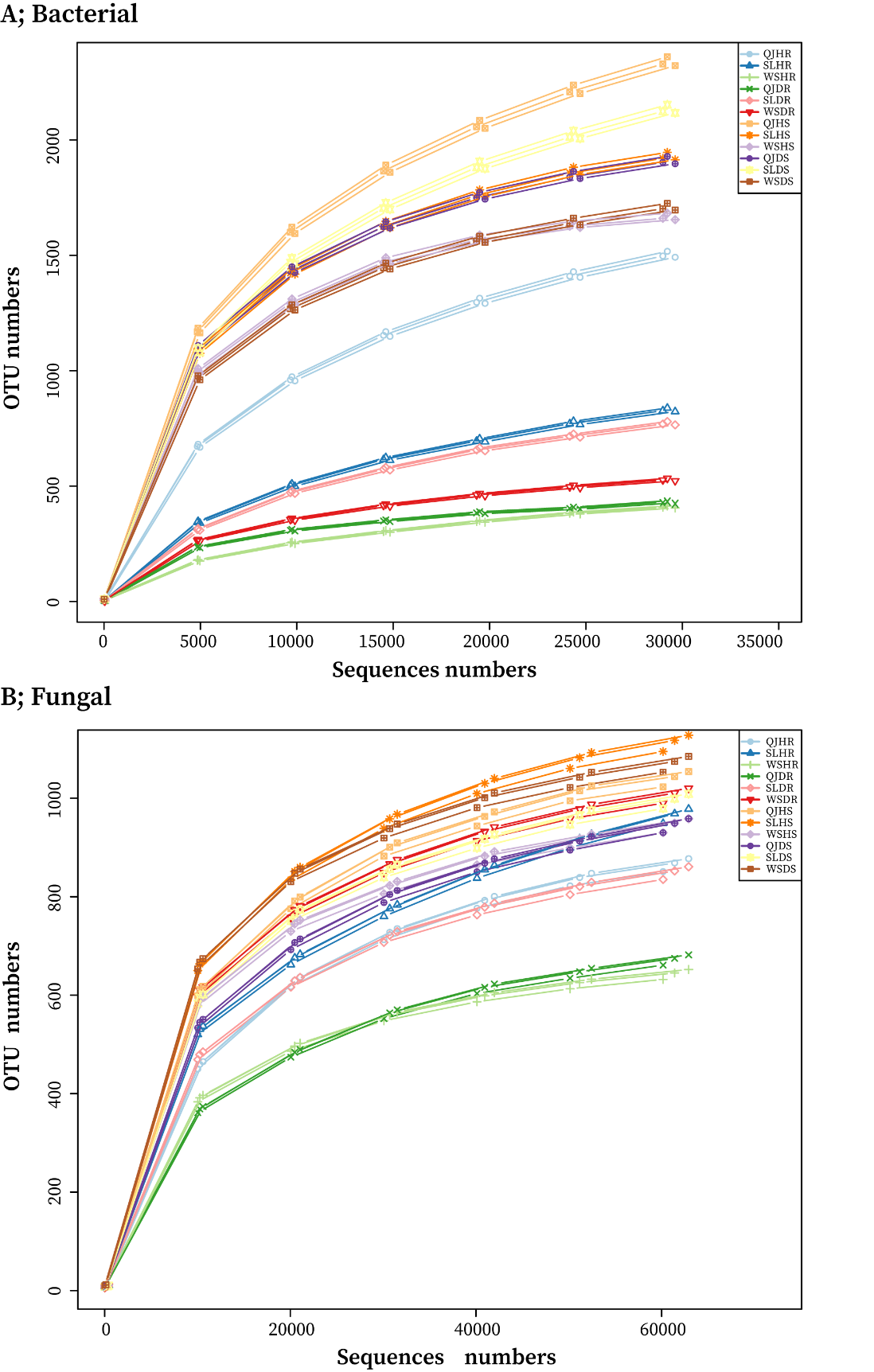
**

**Figure S2:** Rarefaction curves for bacterial (A) and fungal (B) operational taxonomic units (OTUs) generated from all 12 flue-cured tobacco samples collected under three conditions i.e., locations (Qujing, Shilin, and Wenshan), plant components (rhizosphere soil and roots), and sample types (healthy and diseased).


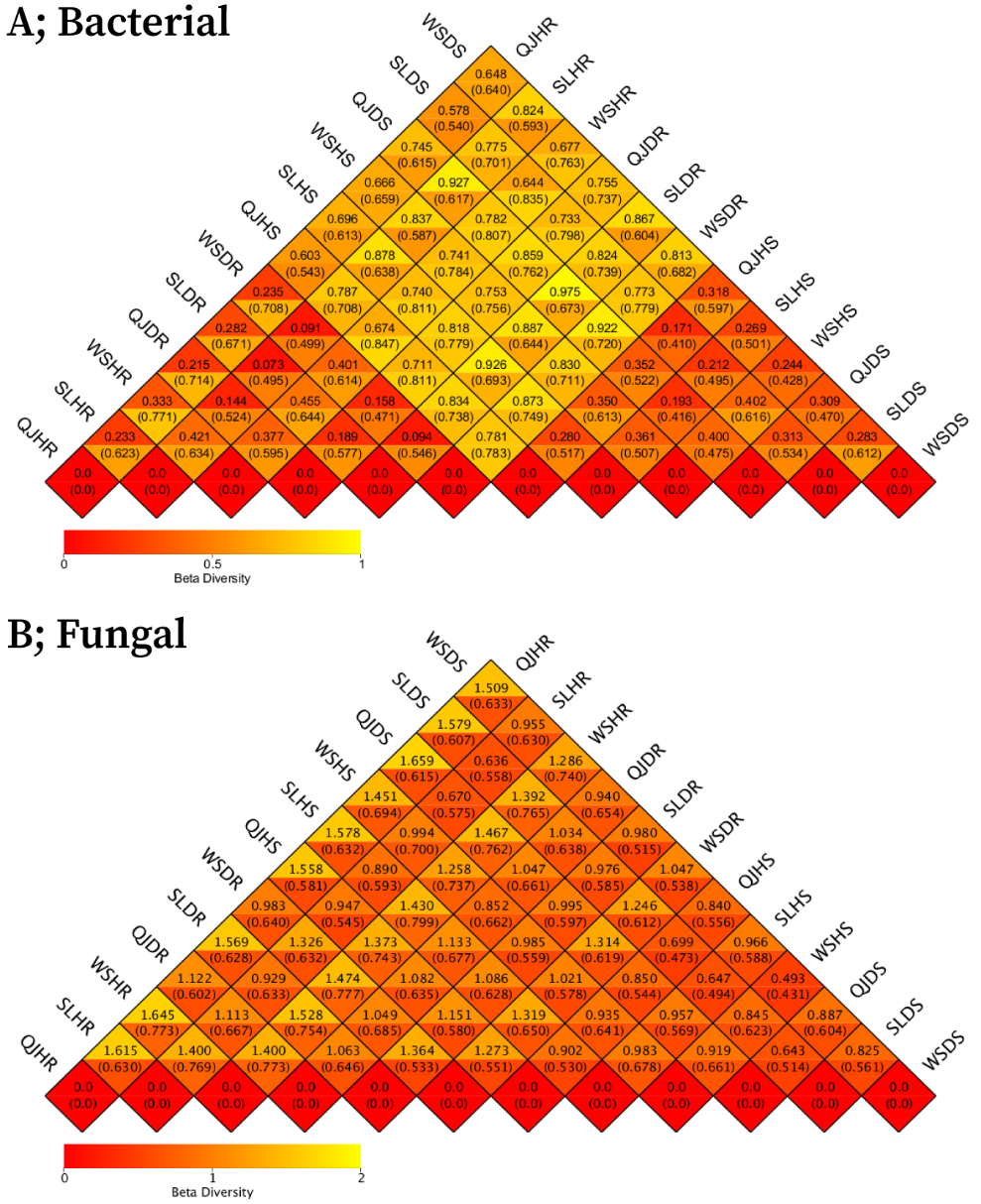


**Figure S3:** Distance heatmap graph of 12 flue-cured tobacco plants samples collected from different locations, sample types, and plant components. Weighted UniFrac and unweighted UniFrac analysis for both bacterial (A) and fungal (B) communities’ analysis. Numbers in grid is the coefficient of dissimilarity between two samples. The smaller the coefficient of dissimilarity, smaller the diversity of species. In the same grid, the upper and lower values represent the distance between weighted UniFrac and unweighted UniFrac, respectively.
